# Endoscopic sinus surgery for foreign body extraction in an adult patient

**DOI:** 10.1002/ccr3.4200

**Published:** 2021-07-06

**Authors:** Arianna Cardella, Andrea Preti, Roberto Gera, Francesco Ottaviani, Francesco Mozzanica

**Affiliations:** ^1^ Department of Otorhinolaryngology Ospedale San Giuseppe IRCCS Multimedica Milan Italy; ^2^ Department of Clinical Sciences and Community Health University of Milan Milan Italy; ^3^ Department of Medicine and Surgery University of Insubria Varese Italy

**Keywords:** endoscopic sinus surgery, nasal foreign body

## Abstract

Foreign bodies are an unusual indication for endoscopic sinus surgery. If outpatient extraction is not possible and acute sinusitis ensues, thorough exploration and extended surgical dissection should be considered to clear the nasal cavities.

## Graphical abstract

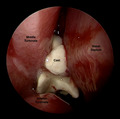

The management of an unusual nasal foreign body is illustrated. A 34‐year‐old man presented to our outpatient clinic after inhalation of liquid cast during preparation of a plaster mask. The foreign body had solidified within the nasal cavities, causing obstruction and headache. Ambulatory removal was incomplete; therefore, endoscopic sinus surgery (ESS) was indicated.

Nasal foreign bodies are extremely common in the pediatric population while being an infrequent presentation for adults.^1^ They are a predisposing factor for acute bacterial rhinosinusitis,^2^ and management of such infection cannot overlook the complete removal of the foreign body.

We illustrate the case of a 34‐year‐old man who developed nasal obstruction, headache, and purulent rhinorrhea following the inhalation of liquid cast during preparation of a plaster mask. Complete ambulatory removal of the solidified cast was not possible, prompting a thorough dissection through ESS (Figure 1) in order to clear all debris and explore the paranasal cavities (Video S1).

**FIGURE 1:**
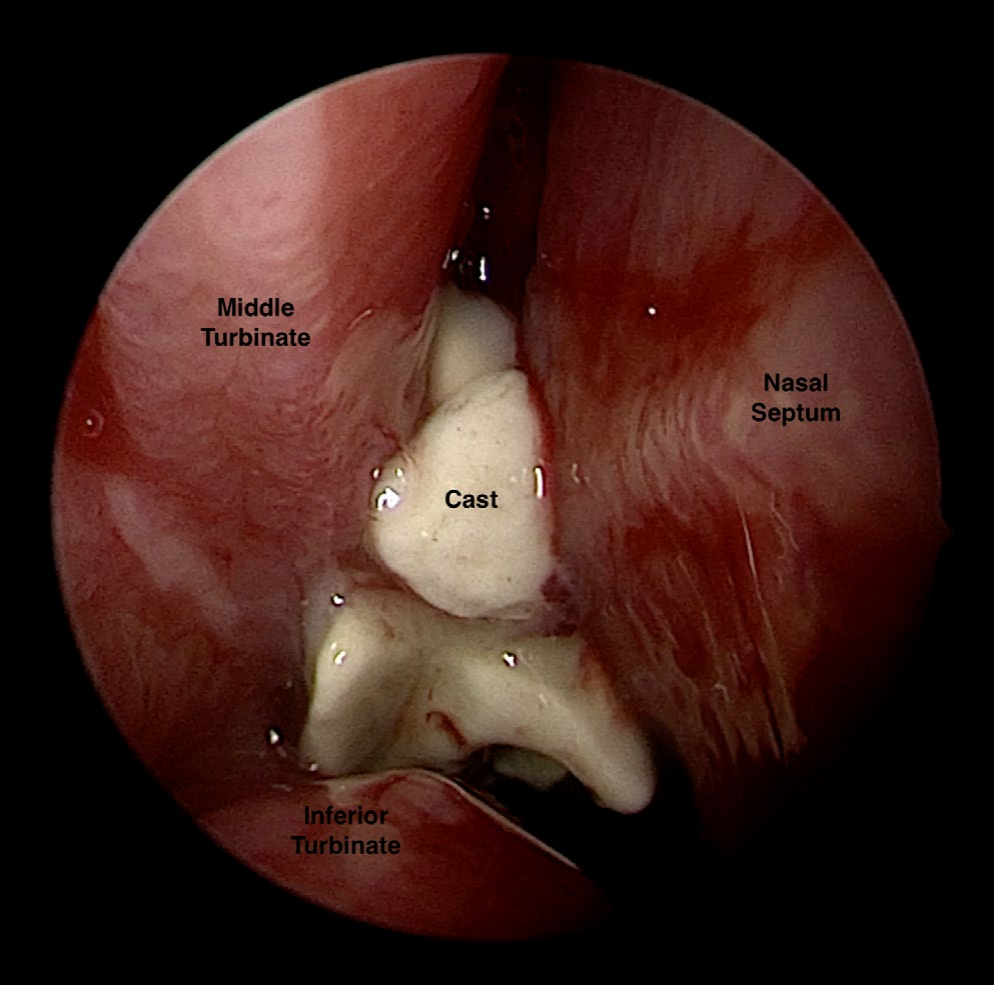
Intraoperative view of the foreign body

## CONFLICT OF INTEREST

None declared.

## AUTHOR CONTRIBUTIONS

AC: involved in a substantial contribution to design of the study; drafting the manuscript; revising the manuscript for important intellectual content, final approval of the version to be published. Agreed to be accountable for all aspects of the work in ensuring that questions related to the accuracy or integrity of any part of the work are appropriately investigated and resolved. AP: involved in a substantial contribution to design of the study; revising the manuscript for important intellectual content, final approval of the version to be published. Agreed to be accountable for all aspects of the work in ensuring that questions related to the accuracy or integrity of any part of the work are appropriately investigated and resolved. RG: involved in a substantial contribution to design of the study; revising the manuscript for important intellectual content, final approval of the version to be published. Agreed to be accountable for all aspects of the work in ensuring that questions related to the accuracy or integrity of any part of the work are appropriately investigated and resolved. FO: involved in a substantial contribution to design of the study; revising the manuscript for important intellectual content, final approval of the version to be published. Agreed to be accountable for all aspects of the work in ensuring that questions related to the accuracy or integrity of any part of the work are appropriately investigated and resolved. FM: involved in a substantial contribution to conception and design of the study; drafting the manuscript, final approval of the version to be published. Agreed to be accountable for all aspects of the work in ensuring that questions related to the accuracy or integrity of any part of the work are appropriately investigated and resolved.

## ETHICAL STATEMENT

This study was carried out according to the Declaration of Helsinki. Patient consent has been signed and collected.

## Supporting information

Video S1

## Data Availability

The data that support the findings of this study are available from the corresponding author upon reasonable request.

## References

[1] Abou‐Elfadl M , Horra A , Abada RL , et al. European annals of otorhinolaryngology. Head Neck Dis. 2015;132:343‐346. 10.1016/j.anorl.2015.08.006 26364542

[2] Fokkens WJ , Lund VJ , Hopkins C , et al. European position paper on rhinosinusitis and nasal polyps 2020. Rhinology. 2020;58(Suppl S29):1‐464. 10.4193/Rhin20.600 32077450

